# Factors Related to Bracket Bond Failure during Orthodontic Treatment: A Single-Centre Single-Operator Study

**DOI:** 10.3390/dj12100300

**Published:** 2024-09-24

**Authors:** Olivier Quinty, Gregory S. Antonarakis, Stavros Kiliaridis, Anestis Mavropoulos

**Affiliations:** 1 Division of Orthodontics, University Clinics of Dental Medicine, University of Geneva, 1205 Geneva, Switzerland; gregory.antonarakis@unige.ch (G.S.A.); stavros.kiliaridis@unige.ch (S.K.); anestis.mavropoulos@unige.ch (A.M.); 2 Department of Orthodontics and Dentofacial Orthopedics, School of Dental Medicine, University of Bern, 3008 Bern, Switzerland

**Keywords:** bracket failure, treatment duration, treatment efficiency, survival analysis, clinical study

## Abstract

This study aimed to investigate the influence of various patient-specific and bracket location-specific factors on bracket survival rates during comprehensive fixed appliance orthodontic treatment. A total of 197 patients (116 females, 81 males; mean age 16.3 years) having completed orthodontic treatment were included in this retrospective cohort study. Patients were treated using stainless steel non-self-ligating brackets, and the treatment duration was 23.7 months on average. The primary outcome was bracket bond failure. Potential predictors for bracket bond failure recorded included age, sex, oral hygiene, treatment duration, and several pre-treatment cephalometric characteristics such as overjet, overbite, and sagittal and vertical skeletal relationships. Factors associated with bracket failure were analysed with Cox regression, and proportional hazard assumptions were assessed using Kaplan–Meier tests. The overall failure rate was 4.4%. Bracket bond failure rates varied among tooth types and seemed to occur more on posterior teeth and on the right side of the arch. Bracket failure was more common in male patients and those with poor oral hygiene. Concerning dentofacial characteristics, bracket failure of anterior teeth was more common in those with an increased overjet and overbite.

## 1. Introduction

During orthodontic treatment with fixed appliances bracket bonding failure may have important consequences for patients and practitioners alike. Most importantly, treatment duration might be longer in patients with bracket bonding failure [1]. A bracket failure has been found to increase the treatment duration by 0.3–0.6 months [2,3]. This may have negative effects on patient compliance and oral hygiene but also increase the risk of developing white spot lesions [4,5] as well as other potential undesirable effects.

Bracket bonding failure may be somewhat inevitable in a number of patients, and in a recent systematic review, the incidence was found to be 0.6–28.3% [6]. There may be, however, factors that increase the risk of bracket failure, and identifying these may help improve patient management and treatment predictability. Previous studies have reported varying factors that may be related to bracket failure [7,8,9], including external patient factors (such as bonding material, bracket material, bonding technique and operator-related factors), patient-specific factors (such as age, sex, oral hygiene, overjet, overbite, facial height, ANB angle) and bracket location (tooth number, maxillary or mandibular arch, anterior or posterior region).

With regard to patient-specific factors, it has been found that adult patients have less bracket failure than adolescents [8,10] and males more than females [7,11]. Oral hygiene does not seem to impact bracket failure, and neither do vertical or sagittal skeletal relationships [12]. It has been found, however, that an increased overbite is associated with increased bracket failure, whereas overjet does not appear to be important [9].

Previous studies looking into bracket failure in relation to bracket location have found that posterior brackets tend to fail more often than anterior brackets [9,13], and brackets in the mandibular arch fail more than in the maxillary arch [8,9,14]. The biggest failure rate has been found on the mandibular second premolars [9,13].

Although external patient factors, both technique-specific and operator-specific, can be controlled by implementing a standardised and low-risk bonding protocol, patient-specific factors are impossible to control since they are inherent to the patient. Knowledge of these would help operators in identifying at-risk patients and perhaps changing either bonding protocols or follow-up regimes for these specific patients.

Existing studies, however, often investigate small samples with multiple operators and this may bias the results obtained. Studies with unique operators and larger sample sizes are thus warranted. The aim of the present study was to investigate the potential influence of various patient-specific and bracket location-specific factors on the survival rate of brackets during comprehensive fixed orthodontic treatment.

## 2. Materials and Methods

The present study was a retrospective cohort study conducted on orthodontic patients having started and completed comprehensive orthodontic treatment with fixed appliances, in a single centre and with treatment having been carried out by a single right-handed operator (with 10+ years of experience in orthodontics). The study was carried out on anonymised data and in compliance with the Declaration of Helsinki.

All patients having started full fixed appliance treatment from 2014 to 2018 and fulfilling the following inclusion criteria were included in the study:

- Patients having been treated by one operator;
- Patients having completed treatment;
- Patients where a direct bonding protocol was used, tubes bonded on the molars and brackets bonded on all other teeth.

The exclusion criteria were as follows:

- Patients with one or both arches having received lingual fixed appliances;
- Patients where indirect bonding was used;
- Previous orthodontic treatment;
- Cleft lip and/or palate or craniofacial anomalies/syndromes;
- Enamel surface abnormalities, including amelogenesis imperfecta, molar–incisor hypomineralisation, or other enamel anomalies of mineralisation, calcification or maturation.

All teeth were bonded with Experience metal non-self-ligating brackets (GC Orthodontics Inc., Alsip, IL, USA) using the same adhesive (Transbond^TM^ XT, 3M Unitek, Monrovia, CA, USA). The bonding protocol was as follows: cleaning of the enamel surface with Zircate prophylactic paste, isolation, etching with 37% phosphoric acid (Reliance orthodontic products, Itasca, IL, USA) for 15 s, primer application (Ortho Solo^TM^, Ormco Corporation, Orange, CA, USA) without air drying or light curing, direct bonding application with 3M Transbond^TM^ XT adhesive, light curing with 3M Unitek Ortholux Luminous Curing Light (wavelength: 440–465 nm; intensity: 1200 mW/cm^2^) for 20 s on tubes and 10 s on other brackets as per manufacturer recommendations.

Patients were seen every six weeks, and throughout the duration of the orthodontic treatment bracket failure was noted in the patient file, along with the teeth in question, by the dental nurse (one of four nurses working with the practitioner). Bracket failures were checked routinely at all regular orthodontic follow-up visits or during emergency visits when the patient came specifically because of bracket failure. Data from teeth with crowns, veneers, vestibular restorations (such as composite or amalgam) or any other non-enamel tooth surface where a bracket had been bonded were excluded, as were teeth that had bands placed instead of brackets. In the case of extraction of teeth for orthodontic purposes, these teeth were excluded from the analysis. At approximately 12 months into treatment, a bracket rebonding session was carried out to improve tooth detailing on teeth deemed to benefit from bracket rebonding to improve positioning. This was not considered as bracket failure but as a new bracket bonding, with the same bonding protocol used.

**Figure 1:**
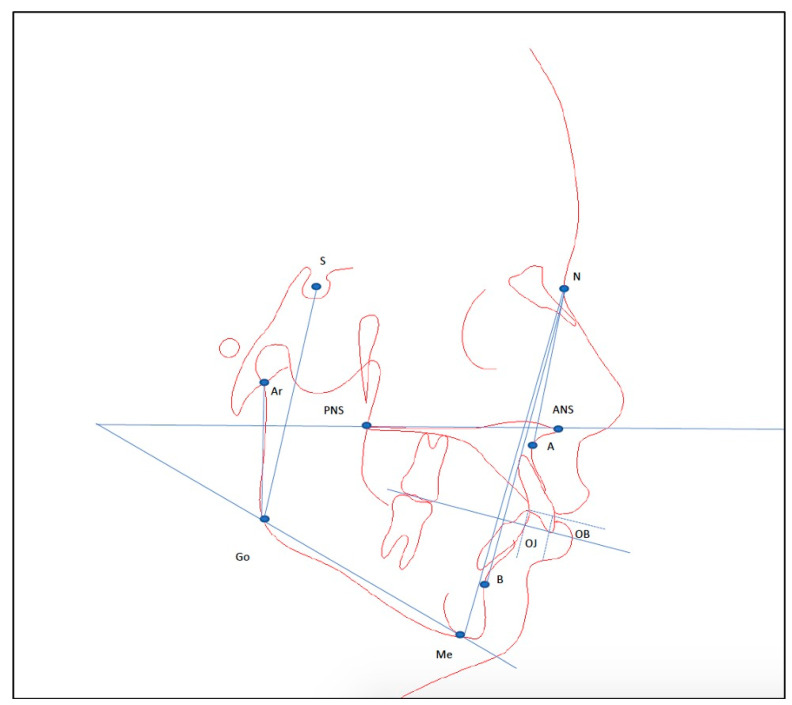
Cephalometric dentofacial characteristics measured on the initial lateral cephalograms of the included patients. Measurements represented in the tracing with blue lines are overjet, overbite, ANB angle, intermaxillary angle (ANS-PNS/Me-Go), gonial angle (Ar-Go-Me), posterior facial height (S-Go), anterior facial height (N-Me). A = A point; ANS = anterior nasal spine; Ar = articulare; B = B point; Go = gonion; Me = menton; N = nasion; OB = overbite; OJ = overjet; PNS = posterior nasal spine; S = sella.

Data were collected from included patients with bracket failure as the primary outcome (tooth number, day of bracket failure, month of bracket failure, duration of time after bonding, first or second bracket failure). Oral hygiene was evaluated on a scale of poor, medium or good, based on the quantity of dental plaque visible on the teeth and around the brackets and reported by the same nurse at each appointment throughout the treatment. Other data collected from each patient file were age, sex, day of bonding, missed appointments, treatment duration, and several pre-treatment cephalometric characteristics (overjet, overbite, ANB angle, intermaxillary angle, gonial angle, posterior facial height, anterior facial height, posterior to anterior facial height ratio) (Figure 1). Cephalometric tracings of pre-treatment lateral cephalometric radiographs were carried out by a single operator, not involved in the treatment of included patients, and blinded to the outcome of bracket failure.

Based on these 14 predictors, a sample size estimation was carried out for a linear multiple regression analysis using an alpha error probability of 0.05, a power of 95%, and a desired correlation coefficient of at least 0.2 (calculation made using G*Power 3.1 for Windows; [15]. This resulted in a minimum sample of 122 patients required.

All statistical analyses were carried out using IBM SPSS Statistics, Version 25.0 (IBM Corp., Armonk, NY, USA). Descriptive statistics and survival analyses were employed to explore the frequency and time to bracket failure and identify factors associated with bracket failure rate per patient. This was calculated with Cox regression and proportional hazard assumptions were assessed using Kaplan–Meier tests. An error of the method analysis was carried out on 20 randomly selected patients (using random.org; accessed on 14 January 2021) by performing the cephalometric analyses again at least 2 weeks after the first measurements. Repeat measurements were assessed for systematic (paired *t*-test) and random error (Dahlberg formula). No systematic error was found, and random error was found to not exceed 0.9 mm for linear and 1° for angular measurements.

## 3. Results

The number of patients initially examined for eligibility was 220, with a total of 23 excluded as they did not comply with inclusion criteria. A total of 197 patients (116 females; 81 males) having undergone and completed comprehensive fixed orthodontic treatment in one centre by a single operator were finally included. The age of the patients at the start of treatment ranged from 9–63 years, with a mean age of 16.3 +/− 10.6 years. The average treatment duration was 23.7 +/− 6.7 months, with a range from 5–47 months. Oral hygiene was judged to be poor in 21.8%, average in 20.5% and good in 57.7% of patients. The number of missed appointments during treatment was 1 for 30.4%, 2 for 14.6%, 3 for 8.4%, and 4 or more for 10.7% of the patients. In total, 35.8% of patients missed no appointments.

**Figure 2:**
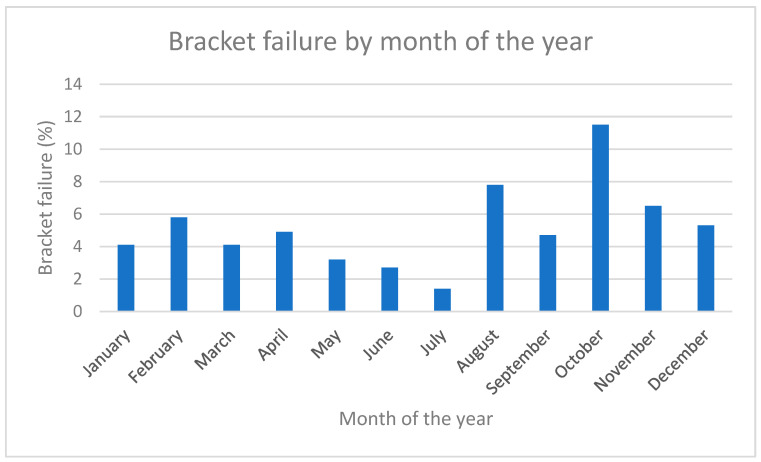
Month of the year when bracket failure was detected.

Regarding the total number of bracket failures per patient, 81 patients had no bracket failure during treatment, while 45 had one, 34 had two, 11 had three, 13 had four, and 13 had five or more bracket failures. The average failure rate per patient was 4.8%, and per bracket (when looking at the totality of brackets) was 4.4%, i.e., approximately 1 in 23 brackets failed. When looking at the month of bracket failure, October had the greatest amount of bracket failures (*p* < 0.001) (Figure 2).

### 3.1. Bracket Location

The failure rate was evaluated for each tooth type, with the highest failure seen for the upper left second premolars and first molars (at 9.3%), while the lowest failure was seen for the upper left lateral maxillary incisor and lower left first premolar (at 0%) (Table 1).

**Figure 3:**
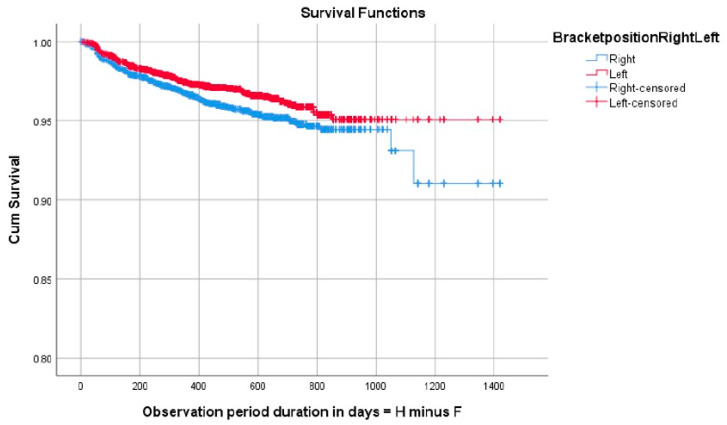
Survival analysis graph showing improved survival in brackets positioned on the left side of the dental arches. Cum Survival = cumulative survival; F = degrees of freedom: H = cumulative hazard rate.

**Figure 4:**
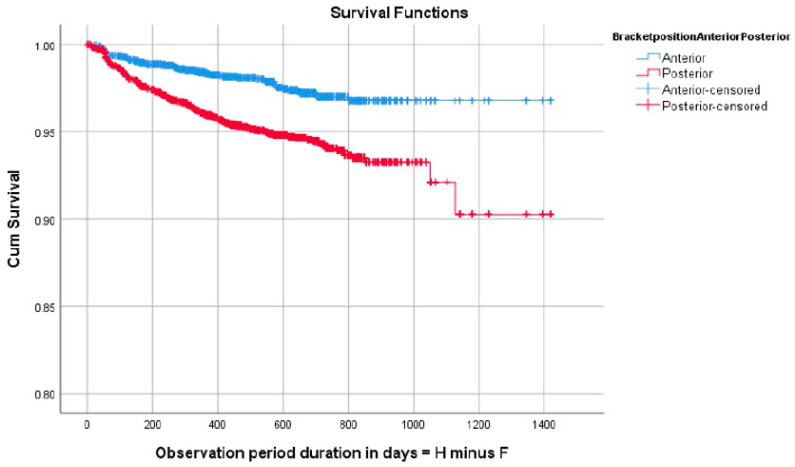
Survival analysis graph showing improved survival in brackets positioned on the anterior segments of the dental arches. Cum Survival = cumulative survival; F = degrees of freedom: H = cumulative hazard rate.

No significant differences were observed when comparing brackets in the maxillary and mandibular dental arches; however, differences were found with regard to failure rate when comparing the right to the left sides of the dental arches, with the right side showing higher failure (*p* = 0.0036) (Figure 3). With regard to the anterior or posterior location in the dental arch, bracket failure was more frequent in the posterior segments (*p* < 0.001) (Figure 4).

### 3.2. Patient-Specific Factors

**Figure 5:**
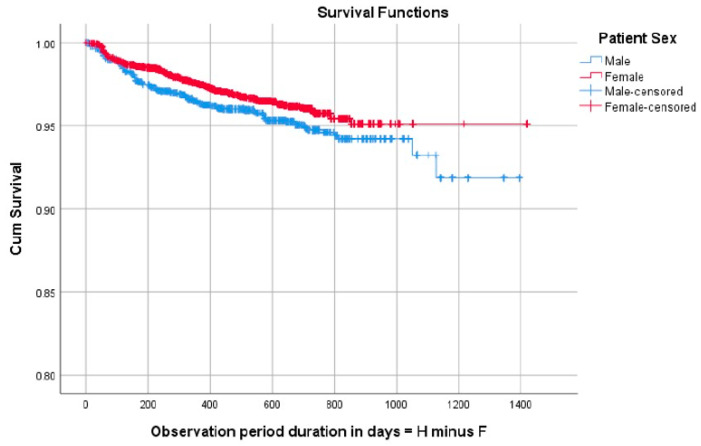
Survival analysis graph showing improved bracket survival in females as compared to males. Cum Survival = cumulative survival; F = degrees of freedom: H = cumulative hazard rate.

**Figure 6:**
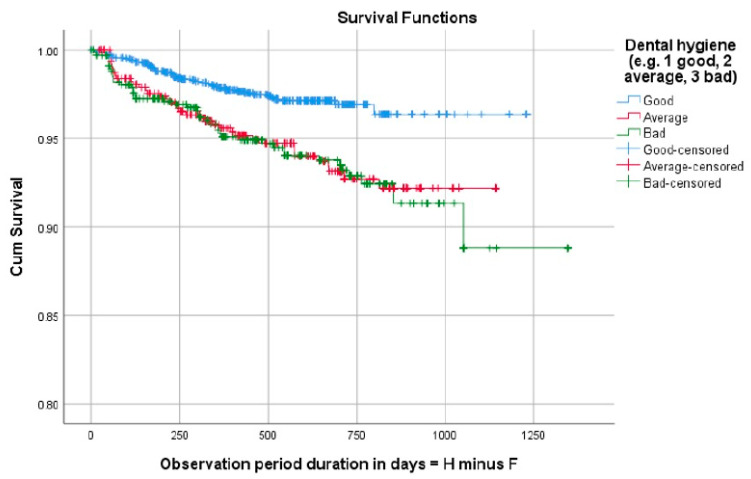
Survival analysis graph showing improved bracket survival in patients with good followed by average followed by poor oral hygiene. Cum Survival = cumulative survival; F = degrees of freedom: H = cumulative hazard rate.

When looking at patient-specific factors in relation to bracket failure, the following results were found. Bracket failure rate was significantly higher in male than in female patients (*p* = 0.0032) (Figure 5). The failure rate was also significantly associated with oral hygiene, whereby those with good oral hygiene showed the best bracket survival while those with poor oral hygiene showed the worst bracket survival (*p* < 0.001) (Figure 6). With regard to age, this was evaluated as a continuous variable and as a dichotomous variable (growing versus adult patients with the cut-off age set at 18), and neither analysis showed associations with bracket failure. Other factors, including the number of missed appointments, bonding day, and length of treatment, were also not associated with bracket failure in the present sample.

The pre-treatment cephalometric variables measured were not correlated to bracket failure in this sample when evaluated as a whole. However, when attempting to split the data into four distinct areas of the dental arch, namely posterior, anterior, upper and lower, significant results emerged. Additional Cox regression analyses were performed using overjet, overbite, the gonial angle, posterior to anterior facial heights, the ANB angle and the intermaxillary angle as covariates. Bracket failure was significantly associated with an overjet >4 mm for maxillary anterior brackets (*p* = 0.048), an overjet of <1 mm for mandibular posterior brackets (*p* = 0.005), and an overbite of >4 mm for mandibular anterior brackets (*p* = 0.011).

## 4. Discussion

The present retrospective cohort study assessed the pattern of bracket failure and potential predictors of bracket failure in a homogeneous sample of patients with fixed orthodontic treatment undertaken by a single operator with a standardised bonding protocol. Results show that the majority of patients who lost brackets lost more than one during treatment, whereas only 23% lost only one bracket. The overall failure rate was 4.4% in this patient population, which was within the range reported in a recent systematic review [6]. The average treatment duration of 24 months in this study might be slightly longer than that found in the literature [6,13], but this did not seem to be related to bracket failure in this sample.

Regarding bracket loss pattern, no significant differences were found according to tooth type which is not in total agreement with other studies [13,16,17]. Posterior teeth nonetheless did seem to show more bracket failure, and the teeth with the highest failure rate were found to be the upper left second premolars and first molars. There was no significant difference in the hazard ratio pertaining to age, although other studies find that younger patients have a higher rate of bracket failure [18], but gender did seem to be a determinant factor. In fact, the bracket failure rate was significantly higher in males than females. This is possibly attributed to the higher masticatory forces exerted by males [19,20] or to the fact that girls are more careful with their appliances [11,21]. The bracket failure rate was significantly associated with the arch position, with posterior teeth having a greater risk of bracket bonding failure. This can be explained by heavier occlusal forces applied on posterior teeth during mastication [22,23], poor moisture control [11,24] or possibly poor adaptation of the brackets due to tooth morphology [11].

In the present study, brackets bonded on the right side of the arch had a significantly higher bracket failure rate than those on the left. This is supported by other studies [25], which find similar differences. These differences may be assigned to masticatory habits, dietary habits, handedness of the operator or exposure to longer periods of moisture when the right-sided brackets are bonded at the end of the bonding procedure.

The present study interestingly showed a significant increase in bracket failure in those with poor oral hygiene. Oral hygiene was evaluated subjectively, simply by eyeballing this parameter on a qualitative scale. This, inevitably, raises questions about its validity, and results should thus be interpreted with caution. Moreover, all patients attending their appointment were instructed to brush their teeth upon arrival with toothbrushes provided to them. This may have introduced further bias in the evaluation of oral hygiene. Nevertheless, this finding may still be relevant and oral hygiene in itself may also be a confounding factor. Patients with poor oral hygiene during orthodontic treatment may also be those who are less inclined to be careful with their appliances and avoid hard and sticky foods. Interestingly, other studies found similar results using a qualitative evaluation of oral hygiene whereby those with poor oral hygiene or with a higher number of remarks regarding their oral hygiene displayed a higher frequency of bracket failure [26,27]. Not all studies agree, however [12].

One of the strengths of this study was the investigation of an array of clinically relevant, potentially predictive, factors influencing the bracket bond failure rate. A further advantage was the large pool of patients treated by one operator with one bracket system and a standardised bonding protocol. Previous studies have reported significant differences between different operators with regard to bracket survival [11,28], and thus, selecting patients treated by a single operator provides this wanted homogeneity. Similarly, bracket failure seems to be dependent on the bonding technique [29], and thus, using a standardised bonding protocol is also beneficial by ensuring homogeneity of the sample.

Despite the advantages mentioned, several limitations were also present. Firstly, the study was a retrospective cohort study with inherent limitations. Some random error cannot be excluded since the dental nurse in charge of taking patient notes may have inadvertently omitted to register an event of bracket failure or made a typing error assigning bracket failure to the wrong tooth. This was a single centre study with the inherent advantages in such a design regarding homogeneity, but the results may inevitably not be largely generalisable. A large multi-centre study would allow for better generalisability of the findings. Finally, the temporal aspect of bracket bonding failure, looking both at the day and the month, may hold some inaccuracy since the incident was recorded on the day of the appointment, not on the day of bracket failure. It must be taken into consideration that the event might have happened days or weeks prior to the appointment, and our finding that October was the month with the highest amount of bracket failure may be biased. The month of October corresponds with the first follow-up appointment for many children after the summer vacation, and thus, this is perhaps when debonded brackets were recorded, even though they may have debonded in reality during the summer break, related to different habits during the summer.

This retrospective cohort study highlights the impact of patient-specific factors on bracket failure during orthodontic treatment. Understanding these risk factors can aid clinicians in identifying patients at higher risk and potentially modifying bonding protocols or follow-up. Further research is warranted to validate and enhance treatment predictability.

## 5. Conclusions

The overall bracket failure rate in the present sample was 4.4%. Bracket failure rates varied between different tooth types and seemed to occur more often on posterior teeth and on the right side of the arch. Bracket failure was more common in male patients and those with poor oral hygiene. Concerning dentofacial characteristics, bracket failure of anterior teeth was more common in those with an increased overjet and overbite. No associations were found with bracket bonding failure and age, number of missed appointments, treatment duration, or any skeletal cephalometric variables.

## Figures and Tables

**Table 1:** Bracket bracket bonding failure per individual tooth. Numbers of brackets exceed 197 because the number includes brackets rebonded for detailing of tooth positioning.

Bracket failures (%)	5.8%	8.4%	6.6%	4.4%	3.6%	0.4%	3.8%	1.5%	0.0%	1.4%	1.9%	9.3%	9.3%	2.7%
Bracket failures (number)	14	19	16	10	11	1	5	4	0	4	4	23	21	6
Total brackets bonded	242	225	242	225	304	233	277	269	231	277	214	248	225	221
Tooth	**17**	**16**	**15**	**14**	**13**	**12**	**11**	**21**	**22**	**23**	**24**	**25**	**26**	**27**
Tooth	**47**	**46**	**45**	**44**	**43**	**42**	**41**	**31**	**32**	**33**	**34**	**35**	**36**	**37**
Total brackets bonded	260	223	250	214	220	223	249	246	231	222	225	249	231	247
Bracket failures (number)	13	18	16	3	4	6	10	13	7	2	0	6	19	3
Bracket failures (%)	5.0%	8.1%	6.4%	1.4%	1.8%	2.7%	4.0%	5.3%	3.0%	0.9%	0.0%	2.4%	8.2%	1.2%

## Data Availability

The data that support the findings of this study are available from the corresponding author upon reasonable request.
